# Volume-sensitive outwardly rectifying chloride channel blockers protect against high glucose-induced apoptosis of cardiomyocytes via autophagy activation

**DOI:** 10.1038/srep44265

**Published:** 2017-03-16

**Authors:** Lin Wang, Mingzhi Shen, Xiaowang Guo, Bo Wang, Yuesheng Xia, Ning Wang, Qian Zhang, Lintao Jia, Xiaoming Wang

**Affiliations:** 1 Department of Geriatrics, Xijing Hospital, Fourth Military Medical University, Xi’an 710032, China; 2 Department of Cardiology, Hainan Branch of PLA General Hospital, Sanya 572031, China; 3 Department of Biochemistry and Molecular Biology, Fourth Military Medical University, Xi’an 710032, China

## Abstract

Hyperglycemia is a well-characterized contributing factor for cardiac dysfunction and heart failure among diabetic patients. Apoptosis of cardiomyocytes plays a major role during the onset and pathogenesis of diabetic cardiomyopathy (DCM). Nonetheless, the molecular machinery underlying hyperglycemia-induced cardiac damage and cell death remains elusive. In the present study, we found that chloride channel blockers, 4,4′-diisothiocya-natostilbene-2,2′- disulfonic acid (DIDS) and 4-(2-butyl-6,7-dichlor-2-cyclopentyl-indan-1-on-5-yl) oxybutyric acid (DCPIB), inhibited high glucose-activated volume-sensitive outwardly rectifying (VSOR) Cl^−^ channel and improved the viability of cardiomyocytes. High glucose induced cardiomyocyte apoptosis by suppressing the autophagic stress, which can be reversed via blockade of VSOR Cl^−^ channel. VSOR activation in high glucose-treated cardiomyocytes was attributed to increased intracellular levels of reactive oxygen species (ROS). Taken together, our study unraveled a role of VSOR chloride currents in impaired autophagy and increased apoptosis of high glucose-exposed cardiomyocyte, and has implications for a therapeutic potential of VSOR chloride channel blockers in DCM.

Hyperglycemia leads to an increase in oxidative stress by exacerbating glucose oxidation and mitochondrial generation of reactive oxygen species (ROS), resulting in DNA damage and accelerated apoptosis^1^,^2^. Hyperglycemia and diabetes mellitus are intimately linked to an increased prevalence of heart failure^3^,^4^,^5^. Nonetheless, antioxidant therapy showed limited effects in the treatment of diabetic cardiac complication, particularly diabetic cardiomyopathy (DCM) and heart failure in humans^6^. This prompts the necessity for a better understanding of the mechanisms underlying the role of ROS accumulation in hyperglycemic cardiotoxicity and cardiac injury.

Autophagy is a highly conserved catabolic progress in which cells generate energy and metabolites by digesting and recycling their own organelles and macromolecucles^7^,^8^. Ample evidence has suggested an essential role for autophagy in the regulation of cardiac structure and function^9^. Hyperglycemia-induced autophagy may contribute to cardiotoxicity in hyperglycemia and diabetes although the precise role of autophagy in DCM has yet to be determined. Upon the initiation of autophagy, LC3 is processed from LC3-I (apparent molecular weight is 16 kDa) to LC3-II (14 kDa)^9^. The levels of LC3-II are proportional to the number of accumulated autophagosomes^10^. SQSTM1/p62 is a polyubiquitin binding protein that is degraded by autophagy, displaying an inverse relationship in level with the autophagic activity^11^,^12^. The lysosomal inhibitor bafilomycin A1 (BAF) is capable of blocking fusion between autophagosomes and endosomes–lysosomes, a process commonly referred to as the maturation or degradation^13^. Autophagy has been demonstrated to participate in the regulation of cardiac hemostasis under both physiological and pathophysiological conditions. Up-regulated autophagy is expected to antagonize ventricular hypertrophy by promoting protein degradation during the transition from hypertrophic cardiomyopathy to heart failure^14^. However, excessive autophagy may also lead to abnormal removal of intracellular protein aggregates, resulting in oxidative stress, reduced ATP production, collapse of cellular catabolic machinery, loss of cardiomyocytes and ultimately cell death^15^.

Accumulating evidence has demonstrated that apoptosis may serve as an essential step in the pathogenesis of a wide variety of cardiovascular diseases^7^,^16^. Nonetheless, the precise mechanism involved in hyperglycemia-induced cardiovascular diseases has not been well identified, which has hindered the development of advance of effective therapeutic strategies in these comorbidities. Normotonic cell shrinkage because of disordered volume regulation, namely apoptotic volume decrease (AVD), is an early prerequisite to apoptosis^17^. The volume-sensitive outwardly rectifying anion channel (VSOR) is known to be involved in a variety of physiological processes including cell volume regulation, cell proliferation as well as cell turnover involving apoptosis^1^. Our previous study has demonstrated that volume sensitive chloride channel specially played an important role in cardiomyocyte apoptosis^18^,^19^. A VSOR blocker (phloretin or NPPB) was found to suppress AVD and caspase-3/7 activation^20^. It was thus expected that VSOR chloride channel participated in apoptosis via mitochondrial or death receptor pathway or ER stress^19^,^21^,^22^. However, the precise mechanisms underlying VSOR chloride channel-induced AVD in the context of hyperglycemia is still unknown. In the present study, we probed the involvement of VSOR Cl^−^ channel in hyperglycemia-induced apoptosis of CMs, and found that VSOR chloride currents promote apoptosis through inhibition of autophagy. These findings have implications for development of a new potential therapeutic strategy for DCM focusing on VSOR chloride channel.

## Results

### High glucose activates volume sensitive chloride currents in cardiomyocytes

**Figure 1:**
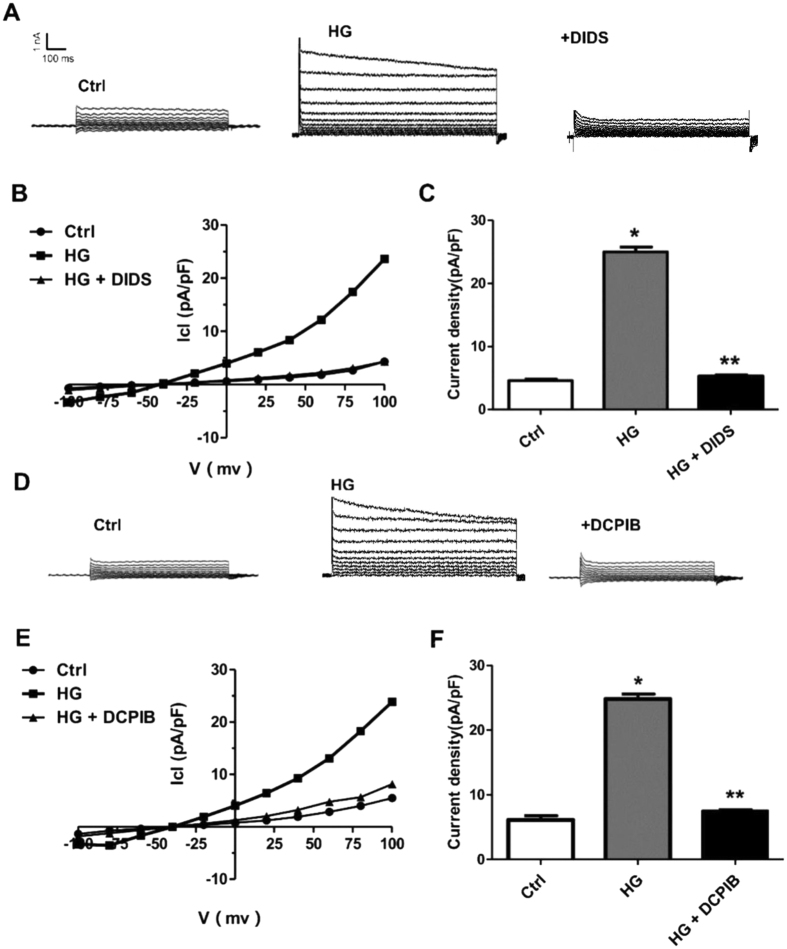
Increased VSOR Cl^−^ currents in high glucose-exposed cardiomyocytes. (**A**) Negligible background Cl^−^ currents were recorded under isosmotic solution (Ctrl). High glucose (HG, 40mM)-induced Cl^−^ currents exhibit representative properties of VSOR Cl^−^ currents, which were inhibited by adding DIDS (500 μM); n = 5 for each group. (**B**) Corresponding current-voltage (I–V) relationship for the mean current densities of cells subjected to indicated treatments. (**C**) Current densities at +100 mV from. (**B**) *P < 0.05 v.s. Ctrl; **P < 0.05 v.s. HG, n = 5. (**D**) Cl^−^ currents recorded under isosmotic solution (Ctrl), HG alone or HG and DCPIB (10 μM) treatment. n = 5 for each group. (**E**) Corresponding current-voltage (I–V) relationship for the mean current densities of cells subjected to indicated treatments. (F) Current densities at +100 mV from (**E**). *P < 0.05 v.s. Ctrl; **P < 0.05 v.s. HG, n = 5.

To examine if volume-sensitive chloride channel is involved in hyperglycemia-induced cardiomyocyte injury, we evaluated the effect of high glucose on VSOR Cl^−^ currents. The whole-cell patch clamp was employed to directly record the VSOR Cl^−^ currents. Cardiomyocytes incubated with high glucose (40 mM; 320 mOsm/L) in extracellular solution switched on a large whole-cell current of I_Cl, Vol_ within ~15 minute (Fig. 1A). The currents were outwardly rectifying with a time-dependent inactivation at +80 and +100 mV (Fig. 1B). Phenotype properties of chloride currents were also recorded (Fig. 1A,D). Compared with control groups, high-glucose stimulus elevated the chloride currents density (Fig. 1C, F). The currents were outward rectifying with a time-dependent inactivation at +100 mV (Fig. 1A,B,D,E). In addition, high glucose-induced currents were significantly inhibited by the VSOR blocker DIDS (Fig. 1A, 78.63 ± 2.32%, n = 5, p < 0.05) and DCPIB (Fig. 1D, 82.56 ± 4.82%, n = 5, p < 0.05). The inhibition was reversed upon washout with hypotonic solution. I_Cl, glucose_ was induced by increasing the extracellular glucose concentration from 5.5 to 40 mM, and stable currents developed within ~15 min. Addition of 500 μM DIDS reversibly inhibited the current measured at -100 and +100 mV by 8.25 ± 1.08% and 78.63 ± 2.32%, respectively (Fig. 1B). The properties of hyperglycemia-related current changes showed that hyperglycemia induces VSOR Cl^−^ currents in cardiomyocytes (Fig. 1A,D).

### VSOR Cl^−^ channel blockers can reverse apoptosis of high glucose-exposed cardiomyocytes

**Figure 2:**
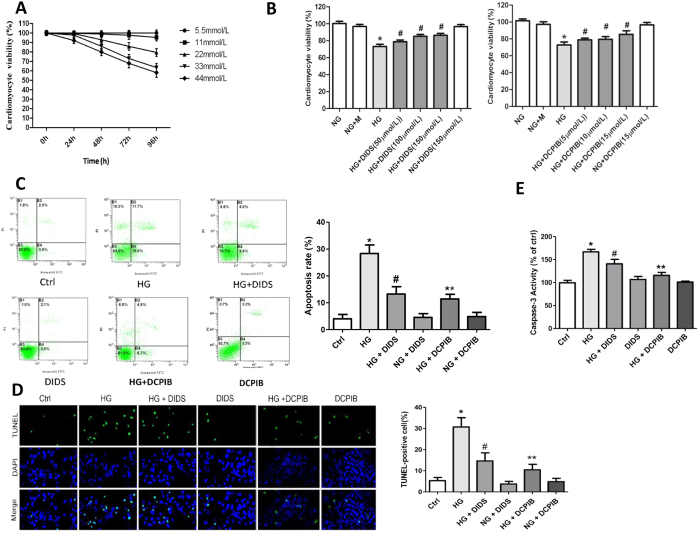
VSOR Cl^−^ channel blockers inhibited cell damage and apoptosis in high glucose-exposed cardiomyocytes. (**A**) MTT-assays of cardiomyocytes exposed to indicated concentrations of glucose. n = 12. (**B**) MTT assays of cardiomyocytes incubated with or without high glucose (HG), DIDS (100 μM) and DCPIB (5 μM). *P < 0.05 v.s. ctrl; ^#^*P *< 0.05 v.s. HG. n = 5. (**C–E**) Cardiomyocytes were treated with DIDS (100 μM) or DCPIB (5 μM) for 72 h under the indicated glucose conditions. Cells were subject to Annexin V/PI staining prior to flow cytometry (FCM) analysis (**C**) and stained for TUNEL. (**D**) The activities of caspase-3 were measured (**E**). Data were expressed as mean ± SD and analyzed by two-way ANOVA (n = 4). Scale bar in (**D**), 200 μm. *P < 0.05 v.s. ctrl; ^#^*P *< 0.05 v.s. HG. n = 5; **P < 0.05 v.s. HG.

Our data presented above suggested a greater increase in VSOR Cl^−^ currents in high glucose-treated cardiomyocytes compared with the control group. We next explored if these Cl^−^ currents were involved in high glucose-induced cardiomyocyte damage. Apoptosis occurs as a result of changes in VSOR chloride channel under hyperglycemia, we treated cells a non-selective Cl^−^ channel blocker, DIDS and a selective VSOR Cl^−^ channel blocker, DCPIB prior to assessment of apoptosis. As shown in Fig. 2A and B, high glucose dose-dependently impaired the viability of cardiomyocytes, which was restored by blockade of Cl^−^ channel with DIDS or DCPIB. Flow cytometry and TUNEL staining showed that high glucose exposure caused a significant increase in the rate of cardiomyocytes undergoing apoptosis (Fig. 2C,D). Further treatment of cells with DIDS or DCPIB suppressed high glucose-triggered apoptosis (Fig. 2C, D). Consistently, high glucose incubation promoted the activation of the apoptosis executioner, caspase-3, which was inhibited by DIDS or DCPIB treatment (Fig. 2E). Meanwhile, cell apoptosis was determined by pro-caspase 3 to confirm the apoptosis process (Supplementary Figure 1). Therefore, blockade of VSOR Cl^−^ channel by DIDS or DCPIB reverses high glucose-induced apoptosis of cardiomyocytes.

### VSOR Cl^−^ channel blockers promote autophagy of high glucose-exposed cardiomyocytes

**Figure 3:**
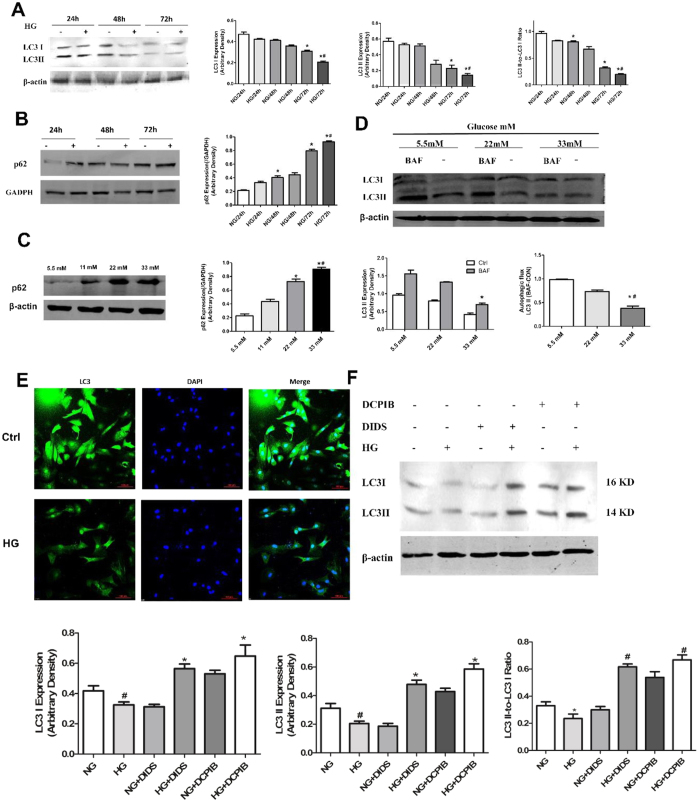
VSOR Cl^−^ channel inhibitors restore cardiomyocyte autophagy impaired by high glucose. (**A–D**) Cardiomyocytes were treated with high glucose (33 mM) for indicated times (24, 48, 72 h) or with indicated concentrations of glucose for 72 h. Western blot analysis and quantitative assays were performed. NG, normal glucose. *P < 0.05 v.s. ctrl (NG, 24 h) and ^#^*P* < 0.05 v.s. NG (72 h) in (**A**) and (**B**). *P < 0.05 v.s. NG (5.5 mM) and ^#^*P *< 0.05 v.s. HG (22 mM) in. (**C**) *P < 0.05 v.s. NG (5.5 mM) and ^#^*P *< 0.05 v.s. HG (22 mM) in (**D**), n = 5. (**E**) Immunofluorescent staining for cardiomyocytes exposed in normal (Ctrl, 5.5 mM) and high (HG, 33 mM) glucose for 72 h. (**F**) Western blot analysis of cardiomyocytes treated with high glucose (33 mM), DIDS (100 μM) or DCPIB (5 μM) for 72 h.

Autophagy may either promote programmed cell death or protect cells from apoptosis in stress conditions. We examined whether high glucose exposure regulated autophagy in cardiomyocytes. As shown in Fig. 3A to C, high glucose time- and dose-dependently impaired LC3 lipidation and p62 degradation, both of which are biomarkers for autophagy. To further validate if the decrease in LC3II was caused by autophagic vesicle (AV) reduction rather than enhanced lysosome degradation activity, we incubated cardiomyocytes with a lysosomal inhibitor bafilomycin A1 (BAF). As a result, BAF markedly increased LC3II levels in cells cultured under normal glucose, but not in cells treated with high concentrations of glucose, suggesting that LCI processing and subsequent lipidation are the major causes of LCII accumulation (Fig. 3D). In addition, immunofluorescent staining showed that high glucose treatment resulted in a significant decrease in LC3II/LC3I levels in cardiomyocytes (Fig. 3E). Rapamycin-induced autophagy activity showed significant increase in cardiomyocytes, and the same effect of up-regulation in autophagy as the VSOR Cl^−^ channel blockers (Supplementary Figure 2F) We next examined whether VSOR Cl^−^ channel blockers protect cardiomyocytes from high glucose-induced apoptosis via autophagy regulation. Indeed, incubation with DIDS or DCPIB markedly increased LC3II/LC3I ratios, suggesting an upregulated autophagic activity by these Cl^−^ channel blockers in high glucose-treated cardiomyocytes (Fig. 3F). We then probed Rapamycin -induced autophagy of cardiomyocytes as a positive control (Supplementary Figure 2A-E).Obviously, there is an up-regulation in autophagy by Rapamycin just as DIDS or DCPIB. Thus, VSOR Cl^−^ channel blockers probably play a protective role in high glucose-exposed cardiomyocytes by restoring the autophagic flux.

### VSOR Cl^−^ channel blockers activate autophagy of cardiomyocytes via suppression of mTOR signaling

**Figure 4:**
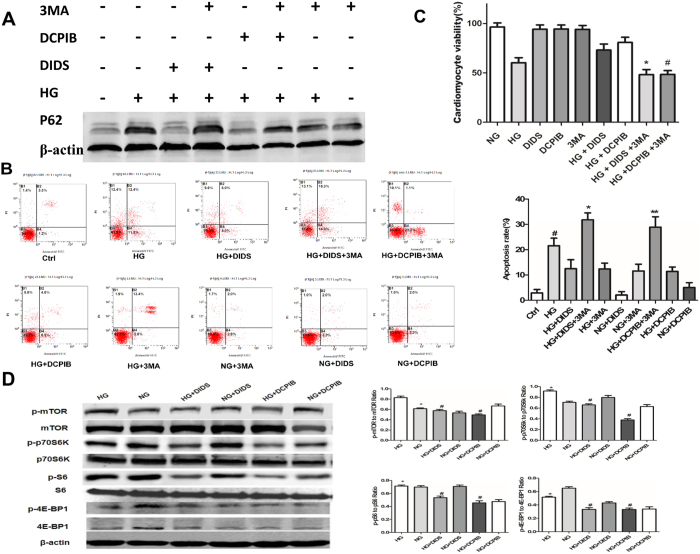
VSOR Cl^−^ channel inhibitors increase cardiomyocyte autophagy via mTOR suppression. Where indicated, cardiomyocytes were exposed to high glucose (HG, 33 mM) and/or treated with DIDS (100 μM) or DCPIB (5 μM) in the presence of vehicle or 3-MA (2 mM). Cells were then subjected to Western blot analysis (**A,D**), Annexin V/PI staining and FCM analysis (B, n = 4), and MTT assays (C, n = 5). ^#^*P *< 0.05 v.s. ctrl (NG); *P < 0.05 versus HG + DIDS group, **P < 0.05 v.s. HG + DCPIB group.

To further test the requirement of autophagy in VSOR Cl^−^ channel blockers elicited protection of high glucose-treated cardiomyocytes, we treated cells with 3-methyladenine (3-MA), an inhibitor of autophagy. As expected, 3-MA significantly inhibited autophagy as revealed by the impeded degradation of p62 (Fig. 4A). More importantly, 3-MA abolished the protective effects of VSOR Cl^−^ channel blockers against high glucose-elicited apoptosis (Fig. 4B). Consistently, MTT assay indicated that 3-MA counteracted DIDS– or DCPIB–induced increase in cardiomyocyte viability (Fig. 4C). We next probed the signaling involved in the autophagic flux triggered by chloride channel inhibitors, and found that the activation of mTOR, the reported classical suppressor of autophagy, was attenuated by VSOR Cl^−^ channel blockers, which was also evidenced by the reduced activation of mTOR complex 1 (mTORC1) targets p70S6K, S6 and 4E-BP1 (Fig. 4D). These data suggest that VSOR Cl^−^ channel blockade protects cardiomyocyte against high glucose damage via suppression of mTOR- signaling and thereby activation of autophagy.

### High glucose–induced ROS production is required for VSOR chloride currents in cardiomyocyte damage

**Figure 5:**
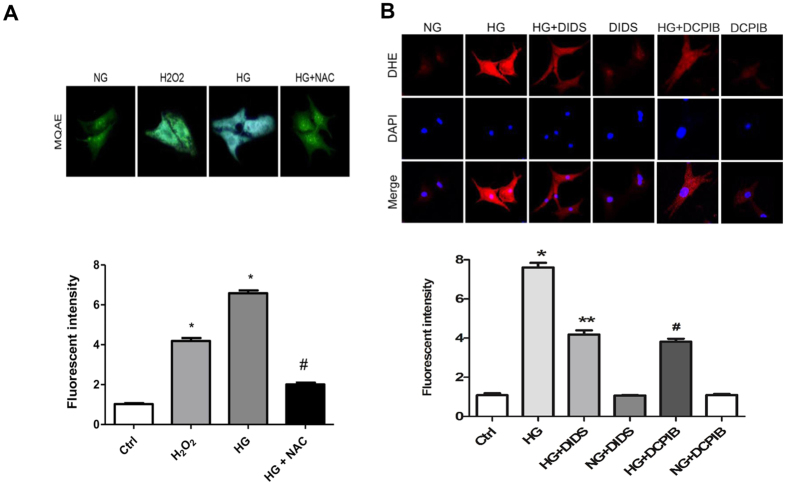
ROS production in high glucose exposed cardiomyocytes. (**A**) Visualization of chloride ion via MQAE staining in cardiomyocytes treated with H_2_O_2_ (500 μM), glucose (33 mM) and a ROS scavenger, NAC (10 mM). *P < 0.05 v.s. NG group; ^#^*P *< 0.05 v.s. HG group. n = 5. (**B**) DHE staining (red) of Effect of cardiomyocytes after normal (Ctrl or NG) and high glucose (HG, 33 mM) exposure for 48 h with or without DIDS (100 μM) and DCPIB (5 μM). Nuclei were counterstained with DAPI. *P < 0.05 v.s. NG group; ^#^*P *< 0.05 v.s. HG group; ^**^P < 0.05 v.s. HG group. n = 5.

Hyperglycemia was reported to impair cell viability via excessive ROS production. To evaluate whether ROS is involved in the activation of VSOR chloride channel in response to high glucose exposure of cardiomyocytes, we performed Dihydroethidium (DHE) staining for superoxide anion (O_2_^−^). Compared with control group, O_2_^−^ was significantly increased in cultured cardiomyocytes after high glucose exposure (Fig. 5B). In addition, treatment of cardiomyocytes with VSOR Cl^−^ channel blockers suppressed high glucose-induced ROS production, suggesting a regulatory role of VSOR Cl^−^ channel in high glucose-induced ROS accumulation (Fig. 5B). We next examined the impact of ROS on the VSOR chloride channel using a chloride ion fluorescent indicator, MQAE, to detect intracellular chloride levels, indirectly reflects chloride channel open or not and inversely related to intracellular chloride ions concentration. Similar to exogenous H_2_O_2_, high glucose treatment of cardiomyocytes dramatically decreased intracellular chloride concentration, which was significantly dampened by a ROS scavenger, NAC, suggesting that ROS is also involved in the regulation of Cl^−^ channels (Fig. 5A).

**Figure 6:**
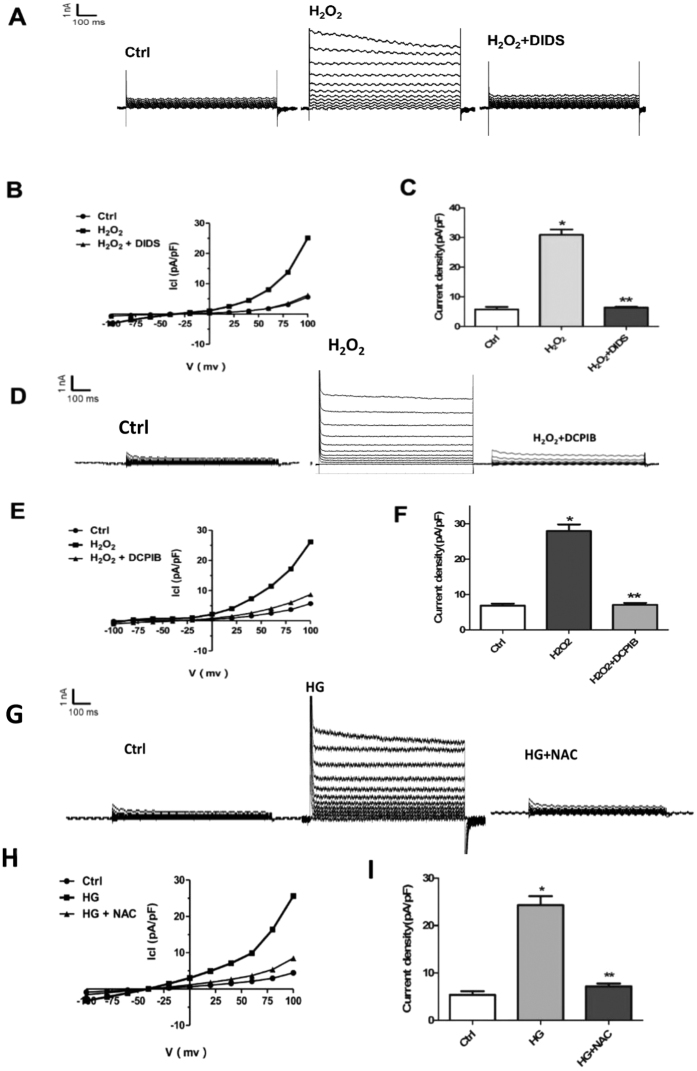
ROS production mediates HG induced VSOR Cl^−^ currents. (**A**) Cl^−^ currents recorded under isosmotic solution (Ctrl), H_2_O_2_ (500 μM) treatment or H_2_O_2_ plus DIDS (500 μM) treatment. n = 5. (**B**) Corresponding current-voltage (I–V) relationship for the mean current densities of cells subjected to indicated treatments. (**C**) Current densities at +100 mV from. (**B**) *P < 0.05 v.s. Ctrl; **P < 0.05 v.s. H_2_O_2_ group, n = 5. (**D**) Cl^−^ currents recorded under isosmotic solution (Ctrl), H_2_O_2_ (500 μM) treatment or H_2_O_2_ plus DCPIB (10 μM) treatment. n = 5 for each group. (**E**) Corresponding current-voltage (I–V) relationship for the mean current densities of cells subjected to indicated treatments. (**F**) Current densities at +10 mV from. (**E**) *P < 0.05 v.s. Ctrl; **P < 0.05 v.s. H_2_O_2_ group, n = 5. **(G**) Background Cl^−^ currents recorded under isosmotic solution (Ctrl). HG (40 mmol/l)-induced VSOR Cl currents exhibiting phenotypic properties of I_Cl, Vol_ (HG). HG-induced VSOR Cl currents were inhibited by the ROS scavenger NAC (10 mM). n = 5 for each group. (**H**) Corresponding current-voltage (I–V) relationship for the mean current densities of isosmotic (⚫), HG(◼) and HG with NAC (▲) conditions. (**I**) Current densities at +100 mV from G. *P < 0.05 vs. Ctrl; **P < 0.05 vs. HG, n = 5.

Next, we explored whether ROS is sufficient for the activation of VSOR chloride channel by recording the VSOR Cl^−^ currents. As shown in Fig. 6A and B, hydrogen peroxide (H_2_O_2_) treatment of cardiomyocytes directly elicited VSOR Cl^−^ currents, which could be inhibited by DIDS (81.56 ± 2.31%, n = 5, *P* < 0.05) or DCPIB (86.53 ± 3.97%, n = 5, *P* < 0.05) (Fig. 6A to F). Conversely, high glucose-induced activation of VSOR Cl^−^ currents was almost completely abolished through scavenging ROS with NAC (10mM, 5–10 min, 97.54 ± 5.23%; n = 5, *P* < 0.05) (Fig. 6G to I). Therefore, ROS production is critically involved in VSOR Cl^−^ channel activation; ROS and VSOR Cl^−^ channel displays a probable reciprocal regulation in high glucose-exposed cardiomyocytes.

## Discussion

In the present study, we demonstrated that high glucose-induced cardiomyocyte apoptosis is accompanied by the outflow of intracellular chloride ions, and the chloride channel blockers DIDS and DCPIB protect cardiomyocytes from apoptosis by maintaining the homeostasis of intracellular chloride ions. Furthermore, we confirmed that high glucose triggered apoptosis by impairing the autophagic flux in neonatal rat cardiomyocytes. These observations suggest that chloride channels are involved in hyperglycemia-induced cardiomyocyte damage, and that blockade of volume sensitive chloride channel and enhancing autophagy may be a potential therapeutic strategy for reducing hyperglycemic cardiotoxicity.

Despite the defined roles of hyperglycemia-related cardiomyocyte apoptosis in cardiovascular diseases such as cardiomyopathy, atherosclerosis and heart failure^4^,^5^, it remains largely uncharacterized how high glucose activates the molecular machineries of apoptosis of cardiac myocytes. The pathogenesis of diabetic cardiomyopathy (DCM) was reported to involve increased reactive oxygen species (ROS) and altered intracellular calcium, ceramides and hexosamines^8^,^23^. In particular, ROS is a key player in these disorders given its established role in high glucose-induced apoptosis^24^,^25^,^26^,^27^,^28^,^29^,^30^. In addition, previous studies have found that the production of VSOR chloride currents resulted in a profound increase in mitochondrial ROS^31^,^32^. We found here that high glucose activation of VSOR chloride channel requires excessive ROS generation in cardiomyocytes, which is consistent with our previous finding that ROS is indispensable for the rising of chloride current and cardiomyocyte ER stress in cardiac contractile dysfunction^21^. Meanwhile, we found that chloride channel blockers reduced high glucose-induced ROS production and apoptosis of cardiomyocytes, suggesting that VSOR chloride channel also acts upstream of ROS in cardiomyocyte damage. Nonetheless, further studies are needed to unravel the precise mechanism underlying the complicated interplay between VSOR chloride channel activation and ROS production in cardiomyocytes.

VSOR chloride channel was reported to promote apoptosis via mitochondrial or death receptor pathway or ER stress^19^,^21^,^22^,^33^, whereas it is unknown whether VSOR chloride channel-induced AVD participates in apoptosis of high glucose-exposed cardiomyocytes. We demonstrated here that high glucose activates the volume-sensitive chloride current, I_Cl, swell_, which displayed outwardly rectifying and voltage-dependent inactivation at large positive voltages. These effects were abolished by the chloride channel blockers DCPIB and DIDS. Swelling-activated Cl^−^ channels serve as an extrusion pathway for Cl^−^, other anions and osmolytes^19^,^34^,^35^. We have shown that the swelling-dependent Cl^−^ current (I _Cl, swell_) in cardiomyocytes had common biophysical and pharmacological properties with the Cl^−^ current induced by cell swelling upon concentrative glucose levels (I_Cl, glucose_), which was reversibly inhibited by DIDS and DCPIB. Since the inhibition is voltage-independent, this is in line with previous studies showing in murine β-cells that the block of I_Cl, swell_ by the HIV protease inhibitors ritonavir and nelfinavir suppressed electrical activity^36^,^37^. It was suggest that this effect contributes to glucose intolerance as a common adverse side effect of HIV therapy^37^. A basal I_Cl glucose_ is active under isotonic, hyperglycemic conditions as evidenced by its sensitivity to the anion channel blockers DIDS, DCPIB (Fig. 1 and ref. 38). These findings suggest that the function of the current to control cell volume and apoptosis is similar to that discussed for I_Cl, swell_ in cardiac myocytes.

Autophagy is reduced in metabolic disorders including obesity and diabetes, which leads to the accumulation of protein aggregates and dysfunctional organelles, and thereby contributes to the pathogenesis of cardiovascular disease^39^. We found here that high glucose reduced the cellular level of LC3-II independently of the lysosome function, and upregulated SQSTM1/p62, a polyubiquitin binding protein inversely related to the autophagic activity. Treatment of cardiomyocytes with DIDS and DCPIB markedly increased autophagy levels and impeded high glucose-induced apoptosis by attenuating the activation of mTOR, a classical suppressor of the autophagic flux. These findings established that high glucose represses the formation of autophagosomes at the early stage of autophagy in neonatal rat cardiomyocytes. Given the dual role of autophagy in affecting the viability of cells in stress conditions, further investigations are needed to dissect the molecular link between autophagy and apoptosis in high glucose-exposed cardiomyocytes. Nevertheless, our results have implications for the clinical treatment of DCM by VSOR chloride blockers that augment the autophagy of cardiomyocytes.

## Materials and Methods

### Reagents and antibodies

D-(+)-Glucose, 4,4′-Diisothiocyanatostilbene-2,2′-disulfonic acid disodium salt hydrate (DIDS), 4-(2-butyl-6,7-dichlor-2-cyclopentyl-indan-1-on-5-yl) oxybutyric acid (DCPIB), 5-bromo-2-deoxyuridine (BrdU), 3-(4,5-dimethylthiazol-2-yl)-2, 3-methyladenine(3-MA), bafilomycin A1 (BAF), 5-diphenyltetrazolium bromide (MTT), n-acetyl-l-cysteine (NAC), and antibody for β-actin, paraformaldehyde, were purchased from Sigma-Aldrich Corporation (St. Louis, MO, USA). Cell Signaling Technology: Anti-p62 c-terminus/SQSTM1 antibody(GP-62C), LC3B(2775). Antibody against pro-caspase-3(2912). 3-methyladenine (Sigma, M9281) in DMEM (HyClone, 11966), and Bafilomycin A1 (LC Laboratories, B-1080) in dimethylsulfoxide (DMSO; Sigma, 472301). Each drug was added to the medium to final concentration as described in each experiment. Dihydroethidium (DHE) was from Molecular Probes (Eugene, OR, USA).Terminal deoxynucleotidyltransferase-mediated dUTP Nick End Labeling (TUNEL) was from Roche Applied Science (Sandhofer Strasse, Mannheim, Deutschland). N-(Ethoxycarbonylmethyl)-6-methoxyquinolinium bromide(MQAE) was purchased from Invitrogen Corporation(USA; E-3101) Molecular Probes, Inc. Cell apoptosis assay by Annexin-V-FITC(fluorescein isothiocyanate)/propidium iodide (PI) staining).

### Neonatal rat ventricular cardiomyocyte culture and high glucose treatment

Neonatal rat ventricular cardiomyocytes were isolated from 0- to 3-d old Sprague-Dawley rat neonates as described previously^19^. After tissue digestion, cells were prepared for 1 h to remove non-myocytes and then plated on gelatinized dishes and cultured overnight in Dulbecco’s modified essential medium (DMEM;GIBCO, 11965–084) with 10% bovine serum. The following day, the cells were washed in phosphate-buffered saline and cultured in DMEM containing 100 Mm of 5-bromo 2′-deoxy-uridine (BrdU; Sigma, B5002). For high glucose studies, cardiomyocytes were cultured for 72 h in glucose free DMEM (GIBCO, 11966) supplemented with 5.5, or 11,22,33 mmol/l of glucose (Sigma, G7021). The osmolarities of all media were made equal to 33 mM by adding different amounts of mannitol (Sigma, M9647). All media contained 100 units/ml penicillin and streptomycin (Sigma, P4333). The study protocol was approved by the Ethics Committee of the Fourth Military Medical University. All experimental procedures used in the study were carried out in accordance with the approved guidelines by the Institutional Review Board of the Fourth Military Medical University.

### Western blot analysis

Cardiomyocytes were lysed by RIPA containing a protease inhibitor cocktail. Electrophoresis and immunobloting were done as described previously^40^. For the densitometry analysis, optical density was measured on the inverted digital images using Image J software. Protein samples were subjected to SDS-PAGE, transferred to polyvinylidene difluoride membrane (Amersham Pharmacia, RPN3031 or Bio-Rad, 162–0177), and then blocked in 5% milk prepared with 0.1% Tween-20 Tris-buffered saline (TBST) solution for 1 h at room temperature. Primary antibodies were incubated overnight at 4 °C in TBS. The membrane was incubated with an appropriate Alexa Fluor^®^ 488 Goat Anti-Rabbit IgG secondary antibody (Abcam, CA11008s New) for 1 h at room temperature in TBST and processed for fluorescence radiation detection using Odyssey Advanced Western Blotting Kit (American Us Limited).

### Determination of cell viability

Cell viability was assessed by MTT assay. Briefly, cardiomyocytes were plated into 96-well culture plates at a density of 5 × 10^4^ /well(100 μl). MTT was added into each well with a final concentration of 0.5 mg/ml, and cells were incubated for 4 h at 37 °C. The formazan crystals were dissolved in DMSO (150 μl/well). The absorbance was detected at 490 nm using a microplate reader (Bio-Rad, USA).

### TUNEL assay

Apoptosis was assayed by TUNEL staining using the *in situ* TUNEL cell death detection kit according to manufacturer’s instructions. In brief, cells were fixed with 4% paraformaldehyde and permeabilized with 0.3% Triton X-100 for 1 h at room temperature, and then washed twice with PBS. Cells were then incubated with the TUNEL assay reaction mixture at 37 °C for 1 h, followed by nuclear counterstaining with DAPI. The number of TUNEL-positive cells in each field was counted and expressed as a percentage of the total number of cells.

### FCM assay

Cell apoptosis assay by Annexin V-FITC (fluorescein isothiocyanate)/propidium iodide (PI) staining The CMs apoptosis was measured using Annexin V-FITC Apoptosis Detection Kit (Invitrogen Corporation), as our established method^21^. Briefly, cultured cells (at a density of 3 × 10^5^ /ml) were washed twice with PBS and incubated in 500 μl binding buffer containing 5 μl of Annexin V-FITC and 5 μl of PI in the dark for 15 min at room temperature. The stained samples were then analyzed on a FACSort flow cytometer within an hour following the manufacturer’s protocol (Beckman Coulter, Fullerton, USA).

### Caspase -3 activity assay

Caspase-3 activity was determined using the caspase-3 activity assay kit (Beyotime, China) according to the manufacturer’s protocol. The assay is based on caspase-3 changing acetyl-Asp-Glu-Val-Asp p-nitroanilide (Ac-DEVD-pNA) into yellow formazan and product pNA^37^. Briefly, cells were collected by centrifugation, washed with cold PBS 3 times, then re-suspended in lysis buffer (100 μl per 2 × 10^6^ cells) and maintained on ice for 15 min. The cells were centrifuged at 18,000 rpm and 4 °C for 15 min. The assay was performed on a 96-well plate by incubating the mixture composed of 10 μl protein sample in cell lysate, 80 μl reaction buffer and 10 μl caspase-3 substrate (2 mM) at 37 °C for 2 h. The caspase-3 activity in cells was quantified using a microplate reader at an absorbance of 405 nm. The protein concentration in the cells was determined using the Bradford protein assay kit (Beyotime, China). The activity of caspase-3 was expressed as the percentage of enzyme activity compared to the control.

### Patch-clamp experiments

Whole-cell path-clamp recording: For voltage-clamp recording, the temperature in all experiments was 23–25 °C. Cells were visualized with an inverted microscope (Nikon) at a magnification of either x200 or x400. Patch pipettes were fabricated from borosilicate glass capillaries using a micropipette puller (P-2000, Sutter Instrument, Novato, CA) with resistance of 3–5 MΩ when filled with pipette solution, Membrane currents were measured with an Axopatch 200B patch clamp amplifier and Digidata1322A (Molecular Devices, Sunnyvale, CA, USA). Pclamp10 software (Version 10.0, Axon) was used for voltage clamp protocols (Fig. 1) and data acquisition. Liquid junction potentials were calculated with JPCalc in pClamp 10 and corrected on-line. For whole-cell recordings, the capacitance transients and access resistance were maximally compensated. To observe the voltage and time dependency of current profile, step pulses were applied from a holding potential of −40 mV to test potentials of −100 to +100 mV in +20 mV increments after attaining each steady state of current action. The amplitude of the current was measured 5 ms after the onset of the step pulses^21^,^33^,^37^. The inhibition percentage of chloride channel inhibitors at the indicated voltage step was determined as follows:(I_Hypo_ − I_Iso_) − (I_Hypo_ + Drug − I_Iso_)/(I_Hypo_ − I_Iso_) 100%.

All chemicals and reagents were purchased from Sigma (St. Louis, MO, USA) unless stated otherwise. For whole-cell recording, the composition of the isotonic bath solution was as followings (in mM): 85 N-methyl-D-glucamine (NMDG), 85 HCl, 10 NaCl, 2 4-aminopyridine (4-AP), 2.5 BaCl_2_, 0.33 NaH_2_PO_4,_ 4 MgCl_2_, 5 Tetraethylammonium-Cl (TEA-Cl), 10HEPES, 5.5 glucose, and 85 mannitol (pH 7.4 adjusted with NMDG-OH, 305 mOsmo/l). The other treatment isotonic bath solution was made by removing some mannitol from the above isotonic solution and adjusted with high glucose, isotonicity was adjusted at the expense of mannitol (pH 7.4 with NMDG-OH, 40 glucose 320 mosm/l).The pipette solution(P1) contains:103 mM CsOH, 103 mM Aspartic acid,25 mM CsCl, 5 mM Mg-ATP, 0.3 mM Na_3_-GTP, 5 mM EGTA, 10 mM HEPES, and30 mM mannitol, (pH7.4 adjusted with CsOH, 295 mOsm/l). The osmolality of all solutions was measured using a freezing-point depression osmometer (OM 802, Vogel, Germany). All reagents except for TTX were first dissolved in dimethylsufoxide (DMSO) as the stock solution, and then further diluted in the corresponding bath solution to a final concentration of DMSO <0.15%. Tetrodotoxin (TTX, 8 μM) and nifidipine (5 μM) were routinely included in bath solutions to block Na^+^ channel and L-type Ca^2+^ channel, respectively. The effect of high glucose on I _Cl glucose_ was studied in whole cell patch clamp experiments. The remaining basal Cl^−^ current was outwardly rectifying and increased upon hypotonic exposure or a raise of the extracellular glucose concentration as previously described^38^,^41^.

### Immunofluorescence for expression of LC3 in cardiomyocytes

Cardiomyocytes were incubated with indicated doses of 5.5 mM and 33 mM for 72 h. Next, cells were fixed with 4% paraformaldehyde for 10 minutes and permeabilized with 0.3% Triton X-100 for 1 h at room temperature. Immunofluorescence assessment of cardiomyocyte expression of LC3 carried out using followed by incubation with the corresponding antibodies in double or triple staining procedures. The LC3-immunolabeled sections with Alexa 488 (green; Molecular Probe) were double stained with cardiomyocyte specific mouse monoclonal anti-α-actin (1:100 in antibody dilution) and rabbit polyclonal anti-LC3 (1:100 in antibody dilution), followed by staining with goat anti-mouse secondary Flour-594 antibody (1:200 in antibody dilution; Invitrogen, Carlsbad, USA) and goat anti-rabbit secondary Alexa Flour 488 (1:200 in antibody dilution; Invitrogen, Carlsbad, CA, USA). Quantitative assessments, including the number of immune-positive dots within cells, were carried out in 20 randomly chosen HPFs (×400) using a multipurpose colour image processor (Nireco, Kyoto, Japan).The sections were observed and images were captured by confocal laser scanning microscopy (Nikon, Japan).

### Intracellular fluorescence measurement of O_2_^−^

Intracellular superoxide was monitored by changes in fluorescence intensity resulting from intracellular probe oxidation according to the previously described method^21^. Cardiomyocyte were loaded with 5 μM dihydroethidium (DHE) for 30 min at 37 °C and washed twice with PBS buffer. Cells were captured using a confocal microscope (Nikon, Japan).

### Statistical analysis

Quantitative data were presented as the means ± SD. Differences between experimental groups were examined by one-way analysis of variance (ANOVA) followed by the Bonferroni post-test using Prism software (GraphPad). *P* < 0.05 was considered statistically significant.

## Additional Information

**How to cite this article:** Wang, L. *et al*. Volume-sensitive outwardly rectifying chloride channel blockers protect against high glucose-induced apoptosis of cardiomyocytes via autophagy activation. *Sci. Rep.* **7**, 44265; doi: 10.1038/srep44265 (2017).

**Publisher's note:** Springer Nature remains neutral with regard to jurisdictional claims in published maps and institutional affiliations.

## Supplementary Material

Supplementary Information
