# Supplementary material for: Volume-sensitive outwardly rectifying chloride channel blockers protect against high glucose-induced apoptosis of cardiomyocytes via autophagy activation
Source: Sci Rep. 2017 Mar 16;7:44265. doi: 10.1038/srep44265 (PMC5353972; doi:10.1038/srep44265)
Supplement: Supplementary Information [file srep44265-s1.doc]

**Supplementary Information**

**for**

**Volume-sensitive outwardly rectifying chloride channel blockers protect against high glucose-induced apoptosis of cardiomyocytes via autophagy activation**

Lin Wang^1,+^, Mingzhi Shen^1,2+^, Xiaowang Guo^1,+^, Bo Wang^1,+^, Yuesheng Xia ^1^, Ning Wang^1^,Qian Zhang^1^, Lintao Jia^3*^, Xiaoming Wang^1*^

^1^Department of Geriatrics, Xijing Hospital, Fourth Military Medical University ,Xi’an 710032, China

^2^Department of Cardiology, Hainan Branch of PLA General Hospital, Sanya 572031, China

^3^Department of Biochemistry and Molecular Biology, Fourth Military Medical University, Xi’an 710032, China

*Correspondence to: Prof. Xiaoming Wang, Department of Geriatrics, Xijing Hospital, Fourth Military Medical University, Xi’an 710032, China; Tel: +86-29-84775543, Fax:+86-29-84775543, E-mail: xmwang@fmmu.edu.cn. Or Prof. Lintao Jia, Department of Biochemistry and Molecular Biology, Fourth Military Medical University, Xi’an 710032, China; Tel: +86-29-84776799, Fax:+86-29-84773947, E-mail: jialth@fmmu.edu.cn.

^+^These authors contributed equally to this work.

Supplementary 1- western blot


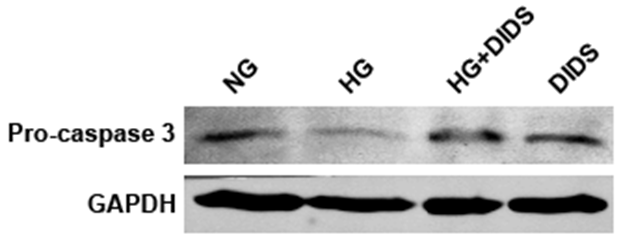


This is original blot with no cropped, no high-contrast.


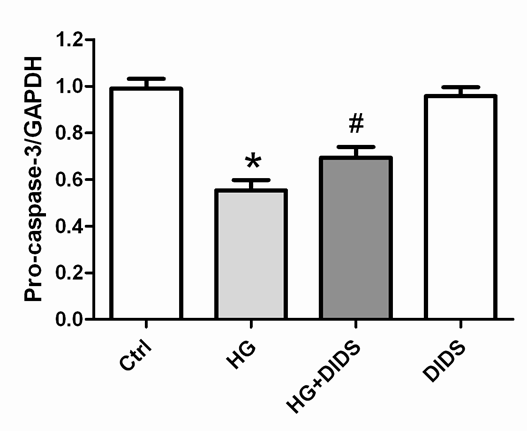


*P<0.05 vs.Ctrl, ^#^P<0.05 vs.HG, n=5

Figure S1: **VSOR Cl^-^ channel blockers can reverse apoptosis and cell injury in high-glucose exposed CMs.** Cardiomyocytes were treated with DIDS(100μM) for 72h under the indicated glucose conditions. Cell apoptosis was determined by pro-caspase 3 (pCasp3,D). The numbers under pro-caspase 3 western blots are fold changes of densities relative to the effect of 5.5 mM glucose( D). Numbers are means with no SD presented (n = 4, ** p*< 0.01 vs. 5.5 mM glucose; *#p* <0.01 vs. 33 mM glucose).

**FIGURE S2-A**


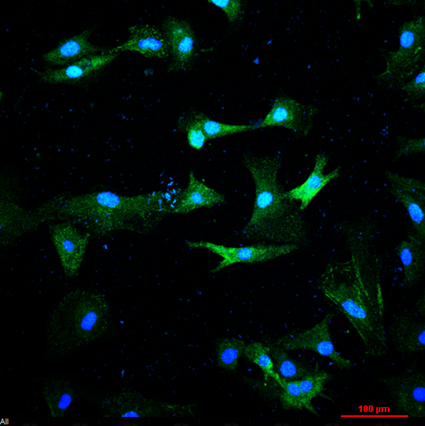


**HG-induced LC3ⅡImmunofluorescent**

**staining dots**

**FIGURE S2-B**

**
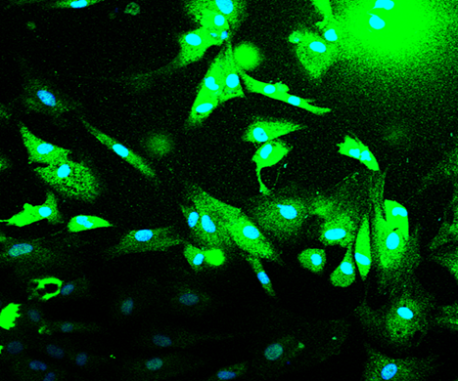
**

**NG-induced LC3ⅡImmunofluorescent**

**staining dots（Ctrl）**

**FIGURE S2-C**


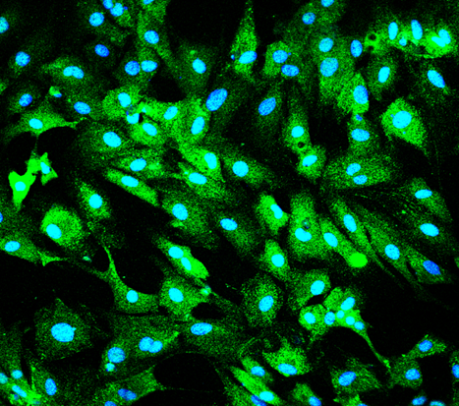


**HG+DIDS-induced LC3ⅡImmunofluorescent staining dots**

**FIGURE S2-D**

**
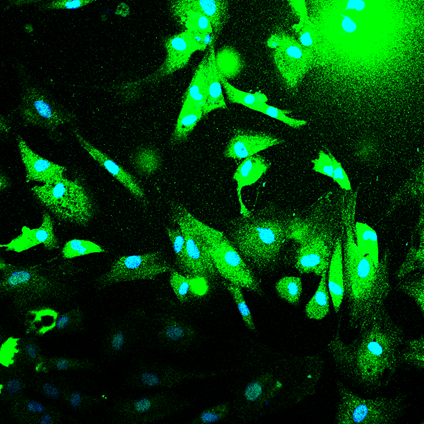
**

**HG+** **Rapamycin -induced LC3ⅡImmunofluorescent staining dots**

**Figure S2 -E**

**
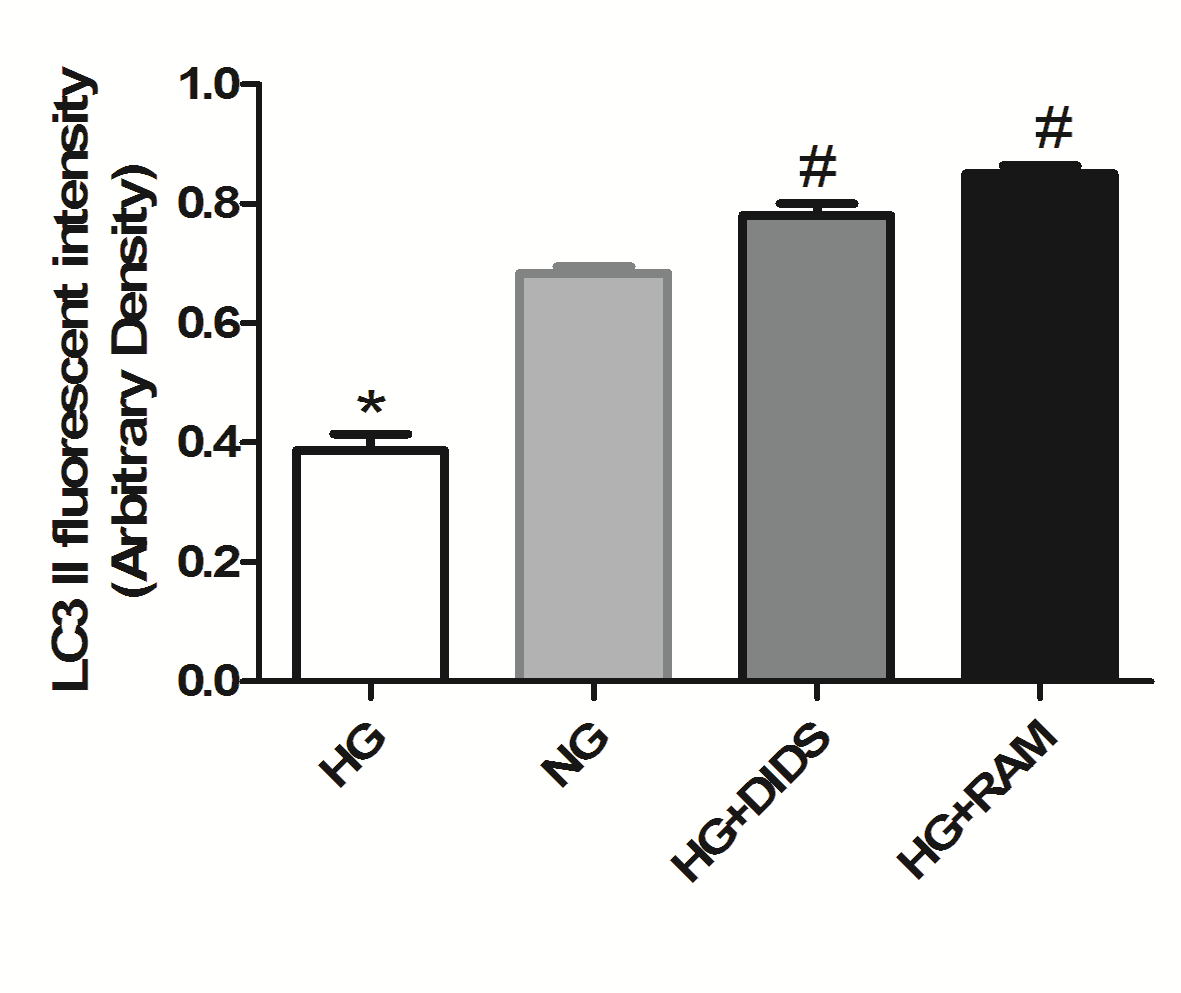
**

**Figure S2A-E** Immunofluorescent staining for cardiomyocytes exposed in normal (Ctrl, 5.5 mM) , HG+DIDS,HG+RAM and high (HG, 33 mM) glucose for 72h. *, P< 0.05 v.s. ctrl (NG, 72h) and ^#^, P< 0.05 v.s. HG (72h)

**Figure S2-F**


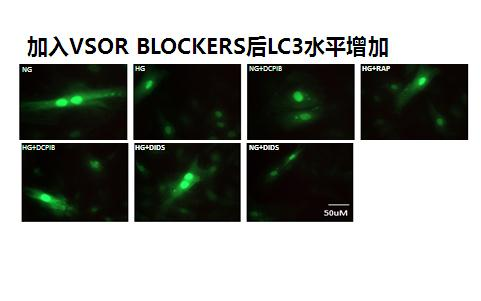


Rapamycin -induced autophagy of cardiomyocytes, and As described above, we’ve included in the revised manuscript a positive control, i.e. Rapamycin -induced autophagy of cardiomyocytes (supplementary Fig S2A-F).
